# Care-Based Collaboration for Regional Sustainability: A Constructivist Grounded Theory Study of Community Nurses and Local Businesses in Japan

**DOI:** 10.7759/cureus.103944

**Published:** 2026-02-20

**Authors:** Akiko Yata, Ryuichi Ohta, Yoshiaki Iwashita

**Affiliations:** 1 Family Medicine, Community Nurse Company, Izumo, JPN; 2 Community Care, Unnan City Hospital, Unnan, JPN; 3 Emergency and Critical Care Medicine, Shimane University, Matsue, JPN

**Keywords:** business model, community nurse, community participation, corporate, general medicine, integrated delivery of healthcare, nursing, public-private sector partnerships, rural health, sustainability

## Abstract

Introduction: Japan’s rapidly aging and depopulating regions face growing challenges in sustaining healthcare, social welfare, and local economies. Community nurses (CNs) have emerged as key actors bridging healthcare, community life, and, more recently, local businesses. However, the processes through which collaboration between CNs and corporations becomes meaningful, operational, and sustainable remain insufficiently theorized. This study explores how CN-corporate collaboration is constructed and how it contributes to regional sustainability beyond short-term economic outcomes.

Methods: A constructivist grounded theory approach was employed. Semistructured interviews were conducted with three senior corporate leaders engaged in formal collaboration with CNs. In parallel, practice-based records, activity narratives, and reflective notes produced by CNs working in small teams within each collaboration were analyzed. Data collection and analysis proceeded iteratively using line-by-line coding, focused coding, constant comparison, memo writing, and dialogic refinement between researchers.

Results: Three interrelated theories were generated. First, corporate engagement emerged from dissatisfaction with reactive, after-the-fact support systems and a reframing of corporate responsibility as inseparable from community survivability. Second, CNs functioned as an implementation-oriented relational infrastructure by receiving delegated implementation authority and mediating across corporate, community, and care systems. Third, collaboration led to a reconstitution of value and sustainability through relational outcomes, including increased resident agency, relational continuity, and community vitality, rather than cost reduction alone.

Conclusion: Collaboration between CNs and local businesses becomes sustainable when care-based, relational practices are delegated, enacted, and valued as investments in community continuity. CNs play a critical role as relational implementation infrastructure, translating preventive care into operational reality and supporting long-term regional and corporate sustainability in aging societies.

## Introduction

Japan is facing a major demographic turning point characterized by rapid population aging, declining birth rates, and a shrinking workforce [1]. These demographic changes have placed increasing pressure on the country’s healthcare and long-term care systems, raising serious concerns about the sustainability of social welfare and the growing mismatch between healthcare needs and available services [2]. In rural and semirural areas in particular, the reduction of healthcare and social resources has made it increasingly difficult to ensure timely, culturally appropriate, and socially embedded care [3,4]. Within this context, there is an urgent need for new, community-oriented approaches that extend beyond institution-centered models of care.

Against this backdrop, community nurses (CNs) have emerged in Japan as licensed nurses who provide preventive, community-based care outside conventional institutional settings [5,6]. Unlike hospital-based nurses, whose roles primarily focus on episodic clinical care, CNs engage in continuous, place-based activities focused on health promotion, early risk identification, and coordination among residents, healthcare providers, and social services. CN activities have expanded in response to population aging, social isolation, and regional depopulation, with more CNs working through local governments, nonprofit organizations, and community initiatives, particularly in rural areas [6,7]. Through their embedded presence in everyday community settings such as workplaces, public spaces, and local events, CNs complement traditional nursing roles by addressing upstream social and health determinants. Through these practices, CNs provide support that extends beyond medical care to include relationship-building, trust-building, and activating residents’ skills and local resources [7,8]. This relational and participatory approach positions CNs not only as care providers but also as mediators, translating institutional healthcare frameworks into forms of support that resonate with local values, practices, and lived experiences.

From a cultural and social perspective, care is not merely a technical intervention but a moral and relational practice embedded in the sociocultural fabric of everyday life [9]. CN activities exemplify this understanding by fostering what Mol conceptualizes as an ecology of care, a network of people, places, and material resources that collectively sustain health and well-being [10]. Through initiatives such as Osekkai conferences and other dialogic community practices, CNs mobilize latent forms of social capital by connecting residents to one another and to external networks, in ways that formal welfare systems alone often cannot achieve [11,12]. These activities highlight the role of CNs in bridging formal and informal care systems while nurturing socially embedded forms of support.

More recently, CN practices have increasingly expanded to include collaboration with local businesses and enterprises, reflecting a broader shift in how care, sustainability, and regional revitalization are understood and enacted. In many communities, local companies face challenges related to workforce aging, employee health, and the long-term viability of regional economies [6]. Through dialogue and joint initiatives with CNs, business leaders begin to engage with health, aging, and care not as external social issues but as integral components of regional sustainability and organizational responsibility [7]. In this process, care is gradually embedded within local economic and social ecosystems, blurring conventional boundaries between healthcare, welfare, and business.

While previous studies in nursing, public health, and community care have described the functions and benefits of community-based nursing, few have examined the processes by which collaboration between CNs and local businesses reshapes the meanings and practices of sustainability and regional revitalization [13,14]. There remains a lack of theory-driven qualitative research that explores how such collaborations are constructed, negotiated, and stabilized through everyday interactions, shared interpretations, and evolving relationships.

To address this gap, the present study adopts a constructivist grounded theory approach to explore the following research question: How does collaboration between CNs and local businesses redefine and implement regional sustainability and revitalization in Japan? By analyzing in-depth interviews with CNs and local business leaders, this study aims to develop a conceptual model that elucidates the processes by which care-based collaboration is embedded in regional contexts. In doing so, the study contributes to theoretical discussions on sustainability, community health, and social innovation, while offering empirically grounded insights into how care can be integrated into local economic and social systems to support resilient and sustainable communities.

## Materials and methods

Study design

This study employed a constructivist grounded theory approach, as articulated by Charmaz and Thornberg, to explore how collaboration between CNs and local businesses redefines and implements regional sustainability and revitalization in Japan [15]. Grounded theory was selected because the study aimed not to test predefined hypotheses but to generate a conceptual model grounded in participants’ experiences, interpretations, and interactions.

From a constructivist perspective, sustainability, care, and collaboration are understood not as fixed entities but as socially constructed through dialogue, negotiation, and practice. This approach allowed for the examination of how meanings related to care, regional responsibility, and sustainability emerged and evolved through interactions between CNs and business leaders within specific regional contexts.

Study setting and context

The study was conducted in several municipalities where CN activities have been continuously implemented, while simultaneously hosting long-standing CN initiatives and emerging collaborations with local businesses. Within this setting, CNs operate across healthcare, welfare, and everyday community spaces, and increasingly engage with local enterprises to address issues such as employee health, workforce sustainability, and regional continuity. This context provided a rich environment for examining how cross-sector collaboration is initiated, negotiated, and embedded over time.

Participants and theoretical sampling

Participants comprised two primary groups: 1) nine CNs engaged in community-based activities and intersectoral collaboration, and 2) three corporate leaders who had participated in formal dialogue or collaborative initiatives with CNs.

Detailed demographic characteristics such as age and sex are not reported to protect participant confidentiality, given the small sample size and the potential identifiability of participants in specific organizational and regional contexts.

Sampling followed the principles of theoretical sampling, whereby data collection and analysis proceeded iteratively. Initial participants comprised CNs and corporate leaders involved in one established CN-business collaboration. As the analysis progressed, additional participants were recruited from two additional CN-business collaborations to elaborate on emerging categories, explore variation in collaborative processes, and examine contrasting organizational contexts. In total, nine CNs and three corporate leaders across three collaborations were included.

Data collection

Data were collected prospectively through semistructured interviews as the primary data source, supplemented by contextual program documents.

Semistructured Interviews

In-depth semistructured interviews were conducted with CNs and local business leaders. Interview guides were flexible and evolved during data collection in response to emerging analytical insights, consistent with grounded theory methodology. Core topics included participants’ experiences of collaboration, interpretations of care and sustainability, perceived changes in roles and responsibilities, and challenges encountered during collaborative processes. Interviews were conducted in Japanese, audiorecorded with participants’ consent, transcribed verbatim, and anonymized before analysis.

Reflective Dialogues and Practice-Based Records

In addition to interviews, this study incorporated practice-based records derived from CNs’ routine dialogic activities and reflective practices in the field. These materials consisted of structured written records documenting CNs’ dialogues with community members, local stakeholders, and business representatives, as well as subsequent reflective notes capturing CNs’ interpretations, uncertainties, and evolving understandings of collaborative processes.

In total, multiple practice-based records were collected across the three collaborations as part of CNs’ routine documentation of field activities and reflective practice. These records included dialogues with community members, corporate representatives, and other stakeholders. Because these records were generated continuously as part of routine practice rather than discrete scheduled sessions, they were analyzed as a corpus of practice-based documentation rather than as individually bounded dialogue events.

These records were systematically compiled and shared among the research team in spreadsheet format to facilitate transparency, comparison, and iterative analysis. The records functioned as contemporaneous accounts of practice, enabling close examination of how meanings of care, responsibility, and sustainability were negotiated and reconfigured through everyday interactions.

Rather than serving as evaluative logs, these reflective records were treated as analytical data capturing the situated sense-making processes of CNs as practitioner-researchers. They were analyzed alongside interview transcripts using constant comparative methods, contributing to category development, refinement of theoretical properties, and validation of emerging interpretations across data sources.

Program Records and Organizational Documents

Program-related documents, including activity descriptions, internal reports, and publicly available materials, were collected to contextualize interview data and clarify the organizational environment in which CN activities and collaborations occurred. In total, the dataset comprised 12 interview transcripts (nine CNs and three corporate leaders), practice-based records generated by nine CNs across three collaborations, and multiple program-related documents describing organizational structures and activities. Program-related documents were used to support contextual understanding rather than as primary analytical data.

Data analysis

Data analysis followed the iterative and concurrent procedures of constructivist grounded theory, whereby data collection and analysis progressed in parallel [16].

Initial Coding

One of the main authors (RO) conducted the initial analysis by repeatedly reading interview transcripts, practice-based records derived from CNs’ field dialogues and reflections, and related program documents. Line-by-line initial coding was performed using action-oriented and process-focused codes to remain close to participants’ language and situated practices. This phase emphasized verbs, interactions, and meaning-making processes, capturing actions such as reframing care as a shared concern, negotiating responsibility across sectors, and translating health-related issues into regional or organizational value.

Focused Coding and Category Development

Codes that were frequent, conceptually rich, or analytically significant were elevated to focused codes and grouped into emerging categories. Constant comparative methods were applied across data sources (interviews, practice-based records, and documents), participant groups, and collaboration stages to refine the properties, boundaries, and dimensions of each category. Throughout this phase, provisional analytic interpretations were documented through memo writing.

Dialogic Refinement and Theoretical Coding

The emerging categories and analytic memos were then examined through iterative dialogue between the two authors (RO and AY). These analytic conversations functioned as a critical reflexive process in which interpretations were questioned, refined, and theoretically elaborated. Through this dialogic engagement, relationships among categories were examined to identify a core category that integrated the developing theory. Analytical attention focused on the processes through which collaboration reshaped understandings of care, responsibility, and sustainability, and enabled their practical implementation within regional contexts. These relationships were ultimately synthesized into a conceptual model illustrating how care-based collaboration became embedded and sustained over time.

Theoretical sensitization

While grounded theory emphasizes theory generation from data, the analysis was sensitized by prior theoretical work in cultural anthropology and care studies. Kleinman et al.’s concept of care as moral experience informed interpretations of how participants framed care within local moral worlds [9]. Mol’s concept of an ecology of care supported analysis of care as emerging through networks rather than isolated interventions [10]. Anthropological perspectives on welfare mediation guided the interpretation of CNs’ roles in locally translating institutional systems into everyday practice [17]. The concept of relational autonomy was used to understand how autonomy was enacted through interdependence and collective decision-making within community and organizational contexts [18]. These frameworks were used as sensitizing concepts rather than as explanatory templates, ensuring that all analytical claims remained grounded in the empirical data.

Reflexivity and analytical rigor

Reflexive memos were maintained throughout the study to document analytical decisions, emerging assumptions, and the researchers’ positionalities, and to critically examine how these shaped data interpretation and theory development.

The first author (AY), as a representative and practitioner of CNs’ activities, brought extensive experiential knowledge of CN practice, including long-term engagement in community dialogues, implementation processes, and collaboration with diverse local stakeholders. AY’s positionality enabled a nuanced interpretation of practice-based data and ensured that emerging theoretical categories remained grounded in the lived realities of CN work. Simultaneously, AY’s proximity to practice required careful reflection on taken-for-granted assumptions within CN activities.

The second author (RO) has been extensively engaged in rural and community-based healthcare in Japan and has completed multiple postgraduate training programs, including master’s and doctoral-level studies in general medicine, public health, and medical education. This background provided sensitivity to structural constraints in rural health systems, interprofessional collaboration, and the social dimensions of care. At the same time, this embeddedness carried the risk of normalizing existing practices or overemphasizing system-level interpretations. To address this, RO explicitly documented moments of analytical tension and uncertainty through reflexive memo writing and subjected preliminary interpretations to critical examination.

Analytical rigor was strengthened through sustained dialogic engagement between the two authors. Differences in disciplinary background, professional roles, and degrees of embeddedness were intentionally leveraged as an analytical resource rather than treated as bias to be eliminated. Through regular analytical discussions, preliminary codes and categories were challenged, refined, and rearticulated, enhancing credibility and theoretical coherence. Iterative comparison across data sources, transparent documentation of analytical decisions, and reflexive interrogation of positionality collectively contributed to the trustworthiness of the analysis.

Ethical considerations

All participants provided informed consent prior to participation. Interview data were anonymized, and no personally identifiable information was included in the analysis. The study adhered to the principles of the Declaration of Helsinki and relevant national research ethics guidelines. Ethical approval was obtained from the Ethics Committee of Unnan City Hospital (approval code: 20250005).

## Results

Participants and interview context

This study analyzed semistructured interviews with three corporate leaders who had established formal partnerships with CNs. All interviewees occupied senior decision-making positions within their organizations and were directly involved in determining the scope, form, and continuation of collaboration with CNs. To protect organizational and participant confidentiality, the names of the organizations are not disclosed. The participating organizations represented three distinct sectors: healthcare-related services, manufacturing, and retail, each operating in rural municipalities experiencing population decline and aging.

Although the organizations differed in industry and organizational structure, the core processes identified in the analysis were consistent across cases. Differences were observed in the operational context and motivations for collaboration; however, these variations contributed to theoretical elaboration rather than fundamentally distinct analytic patterns. Theoretical categories were derived through constant comparative analysis across organizations and regions.

In parallel with the corporate interviews, this analysis incorporated practice-based data from CNs who were actively engaged in each corporate collaboration. In each case, three CNs were primarily involved at the field level, resulting in a total of nine CN practitioners across the three collaborations. These CNs were responsible for the day-to-day implementation of collaborative activities, including community dialogues, coordination with local stakeholders, and ongoing relational work with residents and corporate representatives. Records of their activities, practice narratives, and reflective accounts were included as analytical data to capture how collaboration was enacted, negotiated, and adapted in practice.

The corporate interviewees represented three distinct but complementary positionalities. Interviewee A was a third-generation leader of a regionally embedded healthcare-related enterprise whose organizational identity was historically grounded in preventive health and accessibility. Interviewee B was a fifth-generation executive of an industrial manufacturing firm who approached community engagement as a strategic intervention in response to governance and implementation gaps. Interviewee C was a senior executive in the retail sector, whose business model involved daily, routine contact with community residents and strong place-based embeddedness.

The inclusion of multiple CN practitioners across different corporate contexts enabled analytical triangulation between corporate perspectives and practice-based enactments of collaboration, strengthening the depth and transferability of the findings.

Overview of analytic structure

The analysis yielded three interrelated theories, each comprising multiple subordinate themes. Together, these theories explain how CNs’ regional implementation, enacted through everyday practice and reflective work at the field level and enabled through collaboration with local businesses, contributes to the continuity of local communities and social welfare systems.

The three theories identified were as follows: 1) preconditions for corporate engagement in preventive community implementation, 2) CNs as an implementation infrastructure mediating corporate-community collaboration, and 3) reconstitution of value and sustainability through relational outcomes. Each theory is presented below with its constitutive themes and supported by representative corporate interview excerpts, interpreted in conjunction with CNs’ practice-based activities and reflective records (Table 1).

**Table 1:** Overview of theories, themes, and illustrative quotes derived from a constructivist grounded theory analysis This table summarizes the three interrelated theories, associated themes, analytic descriptions, and illustrative interview quotes derived from a constructivist grounded theory analysis of collaboration between CNs and local businesses in Japan. The table demonstrates how corporate engagement in preventive community implementation emerged, how CNs functioned as a relational implementation infrastructure mediating corporate-community collaboration, and how collaboration led to the reconstitution of value and sustainability through relational outcomes. Interviewee identifiers indicate anonymized corporate participants CNs: community nurses

Theory	Theme	Description	Illustrative quote (interviewee)
Preconditions for corporate engagement in preventive community implementation	Dissatisfaction with reactive, after-the-fact support systems	Corporate leaders recognized the limitations of systems that intervene only after health or social problems escalate	“We keep responding after things have already broken down.” (Interviewee B) CNs observed repeated late-stage support needs in daily community encounters and reflected on structural delays
	Reframing corporate responsibility as community survivability	Corporate responsibility was reframed as inseparable from the survival of local communities rather than philanthropy	“If the town disappears, our business disappears with it.” (Interviewee C) CNs facilitated dialogues linking employee health, aging, and regional continuity
CNs as an implementation infrastructure mediating corporate-community collaboration	Delegation of implementation authority to CNs	Corporations intentionally delegated implementation authority to CNs due to a lack of relational capacity and local expertise	“We can provide money, but we don’t have the people or the know-how.” (Interviewee B) CNs facilitated dialogues linking employee health, aging, and regional continuity
	CNs as a relational and boundary-spanning infrastructure	CNs functioned as intermediaries connecting corporations, residents, and healthcare and welfare systems	“They act as a hub.” (Interviewee A) CNs documented boundary-crossing interactions and iterative adjustments in practice
Reconstitution of value and sustainability through relational outcomes	Redefining outcomes beyond cost reduction	Success was evaluated in terms of relational change, resident agency, and social vitality rather than cost savings	“You can’t measure this by cost savings.” (Interviewee C) CNs reflected on increased resident agency and mutual support emerging over time
	Sustaining community and social welfare through relational continuity	Relational continuity was perceived as essential to the long-term sustainability of communities and businesses	“Keeping the community alive is what keeps our business alive.” (Interviewee A) CNs recorded longitudinal engagement and continuity of relationships across settings

Theory 1: preconditions for corporate engagement in preventive community implementation

This theory explains why corporate actors became receptive to CNs’ preventive and upstream approach despite the absence of immediate financial incentives. The analysis indicates that corporate engagement did not arise solely from abstract recognition of systemic problems, but from convergence between corporate leaders’ dissatisfaction with reactive systems and CNs’ accumulated observations and reflective interpretations derived from everyday practice in the field.

Theme 1.1: Dissatisfaction With Reactive, After-the-Fact Support Systems

All corporate interviewees expressed frustration with healthcare and welfare systems that intervene only after health or social problems have already escalated. This dissatisfaction closely resonated with CNs’ long-standing critique of social systems that “support people only after they become unwell.”

Interviewee B stated: “We keep responding after things have already broken down. At that point, you’re just patching damage.”

Interviewee C similarly observed: “By the time someone shows up in the system, they’re already isolated. That’s not where support should start.”

These corporate perceptions aligned with CNs’ practice-based observations across the three collaborations. CN practitioners working at the field level repeatedly encountered individuals and families only after functional decline, social isolation, or employment-related difficulties had already become entrenched. In their activity records and reflective notes, CNs described recurring patterns in which existing systems responded too late to prevent deterioration, reinforcing cycles of crisis-driven intervention.

Through ongoing dialogue with corporate partners, CNs translated these practice-based insights into shared problem recognition, rendering the limitations of reactive systems visible not only as policy issues but as lived realities affecting employees, customers, and community residents. This convergence functioned as a critical contextual precondition, making CNs’ preventive orientation intelligible and legitimate to corporate actors.

Theme 1.2: Reframing Corporate Responsibility As Community Survivability

Interviewees consistently reframed corporate sustainability as inseparable from the survival of the communities in which they operated. Community engagement was not described as philanthropy or as a form of discretionary corporate social responsibility (CSR), but rather as a rational response to demographic decline, workforce aging, and social fragmentation.

Interviewee C explained: “If the town disappears, our business disappears with it. This isn’t charity-it’s survival.”

Interviewee A linked this logic to inherited organizational values: “Prevention was always part of our philosophy. Supporting people before they decline is just staying true to who we are.”

CNs’ field practices played a central role in concretizing this reframing. By engaging in everyday dialogues with employees, residents, and local stakeholders, CNs surfaced connections between health, aging, work continuity, and regional viability. Their reflective records illustrate how preventive activities, such as informal consultations, relational support, and coordination across sectors, were increasingly interpreted by corporate actors as investments in the social infrastructure sustaining both community life and business operations.

Together, Themes 1.1 and 1.2 demonstrate that corporate engagement with CNs emerged from a convergence of systemic dissatisfaction and a normative reframing of corporate roles, mediated and reinforced through CNs’ ongoing practices and reflective sense-making at the community level.

Theory 2: CNs as an implementation infrastructure mediating corporate-community collaboration

This theory explains how collaboration between corporations and CNs was practically organized and sustained despite asymmetries in expertise, time, and organizational capacity. The findings indicate that collaboration was enabled not through detailed contractual control or standardized intervention models, but through the deliberate delegation of implementation authority to CNs and their subsequent enactment of relational, context-sensitive practices at the field level.

Theme 2.1: Delegation of Implementation Authority to CNs

A central finding was that corporate leaders intentionally delegated implementation authority to CNs. Although corporations could provide financial resources and strategic endorsement, interviewees emphasized their limited capacity to engage in relational work, navigate local dynamics, and adapt preventive activities to heterogeneous community contexts.

Interviewee B stated: “We can provide money, but we don’t have the people or the know-how to do this properly. That’s why we leave it to CNs.”

Interviewee A similarly emphasized restraint: “If we start controlling it, it stops being meaningful. You have to trust the people on the ground.”

From the CNs’ perspective, this delegation translated into substantial discretionary responsibility in practice. Across the three collaborations, CN practitioners working in teams of three per corporate partner made ongoing judgments regarding where, when, and how to engage with residents, employees, and local stakeholders. Their practice-based records document iterative decision-making processes, including modifying planned activities, responding to emergent needs, and recalibrating engagement strategies based on relational dynamics rather than predefined outcome indicators.

Importantly, this delegation was accompanied by a shared rejection of short-term outcome evaluation. CNs’ reflective notes indicate that both CNs and corporate partners came to recognize preventive and relational work as inherently processual, requiring time, trust, and contextual adaptation. This mutual understanding enabled CNs to prioritize relational continuity and responsiveness over measurable short-term outputs.

Theme 2.2: CNs as a Relational and Boundary-Spanning Infrastructure

Interviewees consistently described CNs’ value not in terms of discrete service delivery, but in their capacity to repair disrupted relationships and bridge fragmented systems.

Interviewee A recalled a case involving an isolated older adult: “What mattered wasn’t medical advice. It was someone who stayed with them and helped rebuild trust with professionals.”

Interviewee B described CNs’ intermediary role: “They act as a hub. Information and concerns that never reach us come through them.”

CNs’ practice-based data provide concrete insight into how this boundary-spanning role was enacted. In their field activities, CNs routinely navigated across domains that were institutionally separated, including healthcare, welfare, employment, family life, and informal community networks. They facilitated information flow between residents and corporate actors, translated organizational intentions into locally meaningful practices, and mediated misunderstandings arising from differing temporalities and priorities across sectors.

Through sustained engagement and reflective practice, CNs functioned as a relational infrastructure that stabilized collaboration despite organizational asymmetries. Rather than eliminating these asymmetries, CNs absorbed and negotiated them in everyday practice, enabling corporate-community collaboration to remain adaptive, contextually grounded, and relationally sustainable.

Theory 3: reconstitution of value and sustainability through relational outcomes

This theory explains how corporate actors came to perceive collaboration with CNs as valuable and sustainable, even in the absence of immediate or directly measurable economic returns. The findings indicate that this reconstitution of value emerged through sustained exposure to relational outcomes made visible by CNs’ everyday practices and reflective sense-making in the field.

Theme 3.1: Redefining Outcomes Beyond Cost Reduction

Corporate interviewees consistently rejected cost savings or short-term financial indicators as adequate measures of success. Instead, they emphasized qualitative changes in resident agency, relational density, and the vitality of everyday community life.

Interviewee C stated: “You can’t measure this by cost savings. What matters is whether people start doing things again.”

Interviewee A similarly reflected: “When people move from being helped to helping others, that’s when change becomes real.”

These perspectives are closely aligned with CNs’ practice-based evaluation frameworks. Across the three collaborations, CN practitioners documented shifts in how individuals and groups engaged with their surroundings, such as increased participation in community activities, reengagement with work or social roles, and emergent mutual support among residents. In their reflective records, CNs explicitly contrasted these relational and capability-oriented changes with conventional outcome metrics, emphasizing that such transformations unfolded gradually and were often imperceptible within short evaluation cycles.

Through ongoing dialogue with corporate partners, CNs translated these subtle relational changes into shared understandings of value, enabling corporate actors to recognize outcomes that could not be readily captured through financial or numerical indicators.

Theme 3.2: Sustaining Community and Social Welfare Through Relational Continuity

Corporate leaders viewed CNs’ activities as contributing to the continuity of local communities and social welfare systems, particularly in contexts of population decline and workforce aging.

Interviewee B emphasized long-term regional viability: “If we don’t maintain some level of vitality, the region just gets eliminated.”

Interviewee C connected this to business continuity: “Keeping the community alive is what ultimately keeps our business alive.”

CNs’ practice-based data illuminate how this sense of continuity was enacted and sustained in everyday practice. By maintaining longitudinal relationships with residents, employees, and local stakeholders, CNs functioned as carriers of relational memory across settings and over time. Their records illustrate how continuity was preserved not through discrete interventions, but through repeated encounters, follow-up dialogues, and adaptive support that responded to changing life circumstances.

Through this lens, collaboration with CNs was increasingly understood by corporate actors as an investment in relational continuity, sustaining both community life and the social infrastructure necessary for ongoing corporate operation.

Integrative interpretation of findings

Across the three theories, CNs emerged as an implementation-oriented relational infrastructure that enabled corporations to contribute to preventive community systems without directly managing complex local practices. Corporate collaboration with CNs was sustained when corporate leaders recognized the limitations of reactive support systems, reframed corporate responsibility as intertwined with community survivability, and deliberately delegated implementation authority to CNs.

**Figure 1:**
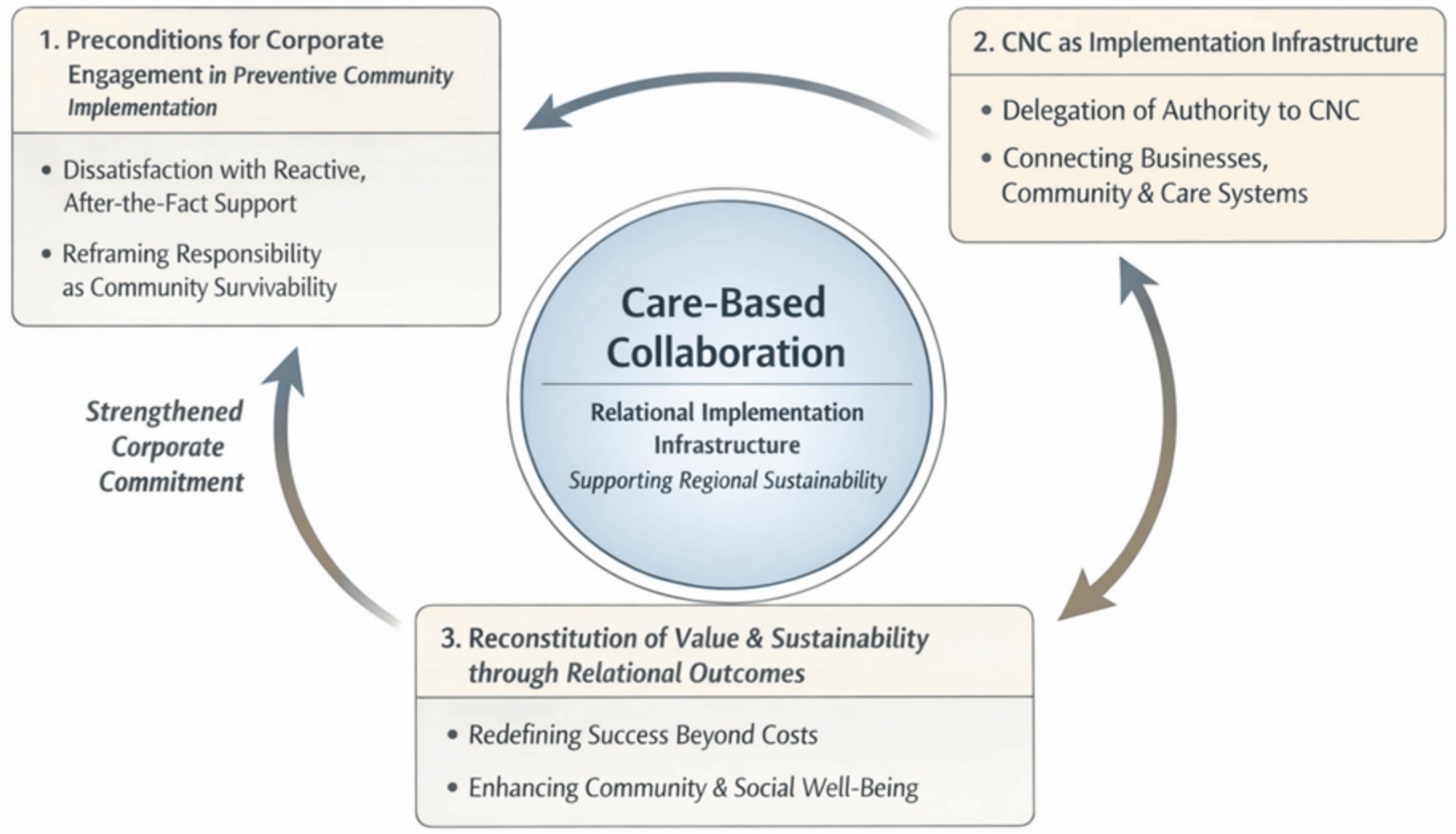
Conceptual model of care-based collaboration between CNs and local businesses This figure illustrates a processual conceptual model derived from a constructivist grounded theory analysis of collaboration between CNs and local businesses in Japan. The model comprises three interrelated theories arranged sequentially and cyclically. First, preconditions for corporate engagement emerge through dissatisfaction with reactive, after-the-fact support systems and the reframing of corporate responsibility as inseparable from community survivability. Second, CNs function as a relational implementation infrastructure, mediating collaboration by receiving delegated implementation authority and bridging corporations, community residents, and healthcare and welfare systems. Third, collaboration leads to the reconstitution of value and sustainability through relational outcomes, including redefining success beyond cost reduction and sustaining community and social welfare through relational continuity. A feedback loop indicates how these relational outcomes reinforce future corporate engagement, supporting regional sustainability through care-based collaboration CNs: community nurses; CNC: Community Nurse Company Image credit: This is an original image created by the author Ryuichi Ohta

At the field level, CN practitioners, working in small teams embedded within each collaboration, translated this delegation into everyday relational work, allowing care-based practices to unfold over time. Through sustained engagement and reflective practice, CNs operationalized a vision of “supporting society from when people are still well,” rendering it visible and meaningful to corporate actors. In doing so, CNs’ activities supported the continuity of local communities, social welfare systems, and the relational foundations of long-term corporate sustainability (Figure 1).

## Discussion

This constructivist grounded theory study elucidates how collaboration between CNs and local businesses in Japan is constructed, mediated, and sustained, and how such collaboration contributes to regional sustainability beyond conventional health or welfare interventions. The findings demonstrate that corporate engagement in preventive community implementation does not emerge primarily from economic incentives, but from a convergence of systemic dissatisfaction and normative reinterpretation of corporate responsibility.

Reframing Corporate Engagement Through Dissatisfaction and Survivability

Consistent with previous critiques of reactive healthcare and welfare systems, corporate participants in this study expressed frustration with interventions that address problems only after they have escalated [14,19,20]. This dissatisfaction served as a critical precondition, rendering CN’s preventive and upstream orientation both intelligible and legitimate [6,21]. Importantly, corporate responsibility was reframed not as philanthropy or CSR in a narrow sense, but as a matter of organizational and community survivability [22,23]. This reframing aligns with emerging literature on place-based responsibility, suggesting that corporate sustainability in depopulating regions is inseparable from the continuity of local social systems.

CNs as a Relational Implementation Infrastructure

A central theoretical contribution of this study is the conceptualization of CNs as a relational implementation infrastructure. Rather than acting as service providers or project managers, CNs mediated collaboration by receiving delegated implementation authority from corporations and translating abstract intentions into contextually embedded practices [24]. This delegation reflected corporate recognition of their own limitations in relational capacity, local knowledge, and long-term engagement [25,26]. In this sense, CNs functioned as boundary-spanning actors who connected corporations, residents, and fragmented care systems, consistent with theories of care as a distributed and relational practice.

This finding extends existing discussions of community nursing and intersectoral collaboration by shifting analytical attention from what services are delivered to how care-based collaboration is implemented and sustained over time [13,21]. CN’s role resembles what Mol describes as an “ecology of care,” in which care emerges through networks of people and practices rather than isolated interventions [10].

Reconstituting Value and Sustainability Through Relational Outcomes

The study further demonstrates that collaboration persisted as corporate actors reconstituted their understanding of value and sustainability. Participants explicitly rejected cost reduction and short-term financial returns as adequate indicators of success, instead emphasizing relational outcomes such as increased resident agency, trust, and community vitality. These outcomes supported relational continuity, which was perceived as foundational to both community well-being and long-term business viability.

This perspective challenges dominant sustainability frameworks that privilege measurable economic outcomes and instead support relational and process-oriented understandings of sustainability [27]. From this perspective, care-based collaboration helps maintain social infrastructure that enables communities and organizations to persist amid demographic decline.

Implications

This study offers several important implications for policy, practice, and theory in aging and depopulating regions. First, the findings suggest that supporting intermediary actors such as CNs may be more effective than promoting direct corporate involvement in community care initiatives. In contexts characterized by relational complexity, fragmented systems, and diverse local norms, corporations often lack the relational capacity and contextual knowledge required for sustained engagement. CNs function as a relational implementation infrastructure that absorbs this complexity, translating corporate intentions into locally meaningful and adaptive practices [6]. Policies that recognize, resource, and stabilize such intermediary roles may therefore enhance the effectiveness and sustainability of intersectoral collaboration.

Second, the study challenges policy frameworks that prioritize short-term, measurable outcomes such as cost reduction or service utilization. Corporate actors in this study came to value outcomes such as relational continuity, resident agency, and community vitality, outcomes that are central to long-term sustainability but difficult to quantify within conventional evaluation cycles. This suggests the need for policy and evaluation frameworks that incorporate relational and process-oriented indicators alongside economic metrics, particularly in preventive and community-based initiatives.

Third, from a theoretical perspective, this study extends discussions of integrated care and public-private partnerships by shifting analytical attention from organizational coordination to implementation processes. Conceptualizing CNs as an implementation-oriented relational infrastructure provides a lens for understanding how care-based collaboration becomes operational and durable in real-world settings. This perspective may be applicable to other contexts in which professional actors mediate between institutions, communities, and markets.

Limitations

This study has several limitations. The sample size was small and focused on corporate leaders who were already positively engaged in collaboration with CNs, which may limit the transferability of the findings to less receptive or more conflictual contexts. In addition, while CNs’ practices and reflections were analyzed in depth through practice-based records, frontline CN practitioners were not interviewed independently, and community residents’ perspectives were not directly captured. Future research should incorporate resident narratives and frontline practitioner interviews to further examine how relational outcomes are experienced across stakeholder groups. Comparative studies across regions with differing demographic, economic, and institutional conditions would also strengthen understanding of the contextual boundaries of the proposed theory. Longitudinal research is needed to examine how relational outcomes and perceptions of value evolve over time and whether they translate into sustained organizational or policy-level change.

## Conclusions

This study provides a theory-driven explanation of how collaboration between CNs and local businesses contributes to regional sustainability in Japan. In response to dissatisfaction with reactive systems, corporate actors reframed responsibility as community survival and delegated implementation authority to CNs. Acting as a relational implementation infrastructure, CNs mediated collaboration and enabled care-based practices to become embedded within local contexts. Sustained through relational outcomes rather than economic metrics alone, such collaboration supports the continuity of community life, social welfare systems, and long-term corporate viability. These findings highlight the importance of care-based, relational infrastructures in addressing the complex challenges facing aging and depopulating societies.
